# Bisacylphosphane oxides as photo-latent cytotoxic agents and potential photo-latent anticancer drugs

**DOI:** 10.1038/s41598-019-42026-y

**Published:** 2019-04-12

**Authors:** Andreas Beil, Friederike A. Steudel, Christoph Bräuchle, Hansjörg Grützmacher, Leonhard Möckl

**Affiliations:** 1 Laboratory of Inorganic Chemistry, ETH Zurich, Vladimir-Prelog-Weg 1, 8093 Zurich, Switzerland; 2 Department of Pharmacology, Toxicology and Clinical Pharmacy, Institute of Pharmacy, University of Tübingen, Auf der Morgenstelle 8, 72076 Tübingen, Germany; 3 Department of Physical Chemistry, Ludwig Maximilian University of Munich, Butenandtstr. 11, 81377 Munich, Germany; 4 Present Address: Department of Chemistry, Stanford University, Stanford, California, 94305 USA; 5 Present Address: ABB Switzerland Ltd., Corporate Research Segelhofstrasse 1K, 5405 Baden-Dättwil, Switzerland

## Abstract

Bisacylphosphane oxides (**BAPOs**) are established as photoinitiators for industrial applications. Light irradiation leads to their photolysis, producing radicals. Radical species induce oxidative stress in cells and may cause cell death. Hence, **BAPOs** may be suitable as photolatent cytotoxic agents, but such applications have not been investigated yet. Herein, we describe for the first time a potential use of **BAPOs** as drugs for photolatent therapy. We show that treatment of the breast cancer cell lines MCF-7 and MDA-MB-231 and of breast epithelial cells MCF-10A with **BAPOs** and UV irradiation induces apoptosis. Cells just subjected to **BAPOs** or UV irradiation alone are not affected. The induction of apoptosis depend on the **BAPO** and the irradiation dose. We proved that radicals are the active species since cells are rescued by an antioxidant. Finally, an optimized **BAPO**-derivative was designed which enters the cells more efficiently and thus leads to stronger effects at lower doses.

## Introduction

**Figure 1:**
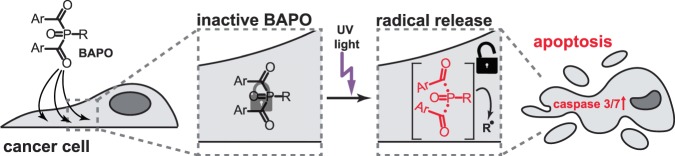
Experimental approach. After cellular uptake, **BAPO** molecules remain inactive inside the cell (left two panels). Upon UV light exposure, they are activated, leading to production of radicals which induces apoptosis via activation of caspase 3/7 (right two panels).

**Figure 2:**
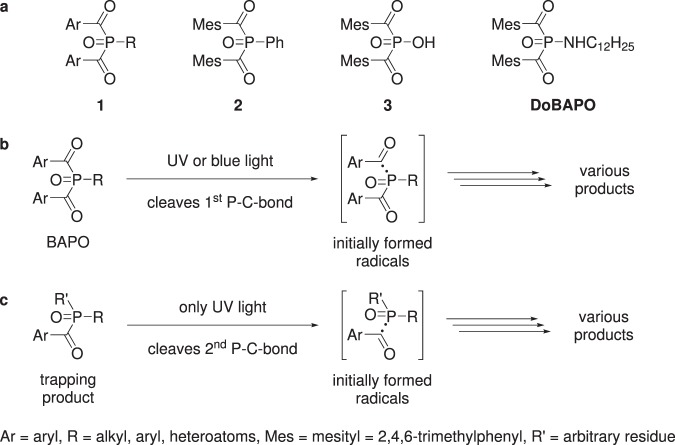
(**a**) Generic structure of **BAPOs** (**1**) and examples for **BAPOs** (**2, 3, DoBAPO**), (**b**) UV or blue light photolyses a generic **BAPO** forming acyl and phosphorous centered radicals, which react to various products. (**c**) The generic trapping product from **b** can be further photolysed by UV light^8,11–15^.

Bisacylphosphane oxides (**BAPOs**, **1**, Figs. 1 and 2a) are a well-established class of photoactive molecules, which form radicals upon light irradiation^1–4^. Thus, they are frequently used as radical photoinitiators in industry and dentistry, e.g. for curing of lacquers or dental fillings^1,2,4^. The economically most important **BAPO** is the phenyl derivative **2**^5^, which is evaluated to be non-toxic according to REACH (see ESI). Another class are hetero-atom substituted derivatives such as bismesitoylphosphinic acid **3**^6–10^ or *N*-dodecyl-*P*,*P*-bismesitoylphosphinic amide (**DoBAPO**). They show a comparable photoreactivity to **2**, yet their solubility in various media is better. Therefore, these derivatives have been used in this study.

The activation of a **BAPO** molecule upon light irradiation forms up to four radicals by homolytical cleavage of the acyl-phosphorous bonds (Fig. 2b,c). A complete activation into four radicals can only be induced by UV light, whereas a single acyl group can be cleaved by blue light^8,11–15^. The minimum reported wavelength required for cleavage is 450 nm^13^ which can also be used in a clinical context^16,17^.

Here, we present a new application for **BAPOs** relevant to medicine, specifically to cancer treatment (see Fig. 1). The presence of radical species in cells induces oxidative stress and eventually apoptosis^18–21^. Hence, substances like **BAPOs** can be regarded as latent cytotoxic agents for the treatment of solid cancers. Besides, non-irradiated **BAPOs** are known to be non-toxic^10,22,23^. As the active agents, i.e. the radicals, are only produced in the respective tissue after irradiation, this class of compounds should cause fewer side effects than standard cytotoxic therapies. Another advantage is that the mechanism of action of **BAPOs** is rather independent of the cancer entity. We therefore consider them as a promising class of molecules for an application in photolatent therapy and investigated their potency as cytotoxic agents.

## Methods, Chemical Syntheses, and Analyses

### General synthetic considerations, materials, and analytics

If not otherwise mentioned, reagents and solvents have been purchased from commercial suppliers. Triethylamine was distilled from calcium hydride prior to use. Solvents have been degassed and purified using an Innovative Technologies Pure Solv solvent purification system prior to use. All syntheses and manipulations except the chromatographic purification of **DoBAPO** have been performed under dry argon under the exclusion of air and moisture using standard Schlenk techniques. All syntheses and manipulation have been performed under the exclusion of light. Bismesitoylphosphinic acid (**3**) has been synthesized according to literature^7^. Human blood plasma has been obtained from sodium citrate (3.2%) blood by centrifugation and stored at −20 °C until used. Informed consent from the individual donating the blood sample was obtained and the sample was taken by a medical specialist.

NMR spectra have been recorded on Bruker 300 and 400 MHz spectrometers. Chemical shifts are reported in ppm relative to SiMe_4_ (for ^1^H and ^13^C) and 85% phosphoric acid (for ^31^P) using the solvent deuterium signals as internal standards. Signal multiplicities are described as singlet (s), doublet (d), triplet (t), quintet (quint), and multiplet (m). If not otherwise stated, ^13^C signals are singlets. Means of 2D NMR methods have been used for signal assignment. Data analysis has been carried out using Bruker TopSpin 3.1.

Elemental analyses have been carried out at the ETH Zürich Mikrolabor.

MALDI-TOF mass spectra were recorded at the ETH Zürich MS department on a Bruker UltraFlex II system.

Single crystal X-ray diffraction measurements were performed on a Bruker Smart Apex2. Data analysis were performed using Bruker Apex2 and Olex2 1.2 software^24^. A crystallographic information file for **DoBAPO** is deposited at the Cambridge Crystallographic Data Centre under CCDC Number 1503437.

Melting points have been determined on a Büchi Melting Point M-560 apparatus.

### Software

For general data visualization and analysis, OriginPro 8G or 9.1G has been used. Images were analyzed using ImageJ. Figures were prepared with CorelDrawX8.

### NaBAPO

Bismesitoylphosphinic acid (**3**) has been synthesized according to literature^7,25^. **BAPO 3** (224.0 mg, 0.625 mmol) is dissolved in NaOH aq. (1.0 M, 6.4 mL). Dilution to 25 mL yields a clear, yellow solution containing 25 mM **NaBAPO**. This stock solution is stored at −20 °C or −80 °C until used. **NaBAPO** has been proven to be stable under these conditions for at least six months.

### DoBAPO

Bismesitoylphosphinic acid (**3**, 1453 mg, 4.05 mmol) is dissolved in dry THF (10 mL) in an argon atmosphere, followed by the addition of dry DMF (0.05 mL, catalytic amount). While stirring at r.t., oxalyl chloride (0.6 mL, 7.0 mmol, 1.7 eq.) is slowly added. After stirring the reaction mixture for 2 h at r.t., all volatiles are removed *in vacuo*. The obtained crude bismesitoylphosphinic chloride (**7**) is dissolved in dry THF (40 mL)^25^. Triethylamine (1.7 mL, 12 mmol, 3 eq.) is added to this orange solution while stirring. Upon the addition of dodecylamine (751 mg, 4.05 mmol, 1 eq.) in dry THF (10 mL), a colorless solid precipitates and the color changes to yellow. After stirring for 30 min at r.t., the reaction mixture is filtered over Celite® and evaporated *in vacuo*. The crude, orange reaction product is quickly purified by column chromatography (100% EtOAc, silica) yielding *N*-dodecyl-*P*,*P*-bismesitoylphosphinic amide (**DoBAPO**) as pale yellow solid (1790 mg, 3.40 mmol, 84%). Single crystals suitable for X-ray diffraction are obtained by slow evaporation of an EtOAc solution. The analytical data of **DoBAPO** are given in the ESI. Figure S1 shows the molecular structure of **DoBAPO** while in Table S1 the crystal data and structure refinement parameters are listed.

For the liposomal formulation, **DoBAPO** is dissolved in 5 µL Tween® 20 per mg substance (1.9 µmol) while heating in an Eppendorf Thermomixer comfort (80 °C, 1000 rpm) until a clear, yellow solution is obtained. While shaking at 80 °C/1000 rpm, addition of PBS in three portions to a final volume of 190 µL per mg leads to an turbid liquid containing 10 mM **DoBAPO** and ca 2.5 v-% Tween® 20. Upon cooling to ambient temperature, the mixture remains unaltered. This formulation is freshly prepared prior to use.

Except for UV/Vis spectroscopy in EtOH without additives, **DoBAPO** always refers to this liposomal formulation when used in experiments.

### UV-Vis spectra and molar extinction coefficients

UV-Vis spectra (see Fig. S2) have been recorded in Hellma QS-110 10 mm quartz cuvettes on an Analytic jena Specord 200 spectrometer using WinASPEC 2.2 software.

Raw absorbance data have been smoothed (Adjacent-Averaging, Points of Window: 5), corrected by subtraction of smoothed values of a solvent blank (PBS or EtOH, respectively) and plotted against the wavelength. Local absorption maxima λ_max_ have been determined to be very similar between the three samples with values of 284 and 286 nm, respectively. Molar extinction coefficients ε (listed in Table S2) have been determined according to the Lambert-Beer law by linear regression of the absorbance values at λ_max_ and at 365 nm (the wavelength applied for **BAPO** activation in *in vitro* experiments), respectively. R^2^ values from the regression analysis are given. Please note that especially the values ε(365 nm) for **NaBAPO** in PBS and **DoBAPO** in EtOH must be regarded as inaccurate due to low absorbances at this wavelength. Furthermore, the extinction coefficients ε of the liposomal formulation of **DoBAPO** must be regarded with care as the Lambert-Beer law is not applicable to colloidal solutions due to light scattering.

### Determination of particles size in liposomal formulation of DoBAPO

Dynamic light scattering (DLS) analyses have been performed on a Malvern Zetasizer nano zs using software version 7.11. A stock solution of **DoBAPO** is diluted to working concentrations (100 µM, 50 µM, 25 µM, 10 µM) with PBS and DLS measurements are performed in triplicates. For each condition, the individual size distributions by number are extracted, averaged and plotted against the particle size. Error bars indicate standard errors from triplicates (Fig. S3). Particles with maxima in size distribution around 35 nm are observed. No influence of the concentration is detected.

### Plasma stability of BAPOs

To determine the stability of **BAPOs** under physiological conditions, the **BAPO** stock solutions used in this study have been incubated with human plasma and DMEM tissue culture medium at 37 °C for 24 h (*vide infra*). The **BAPO** content was determined by ^31^P NMR spectroscopy in comparison with triphenyl phosphate as external standard (*cf*. Fig. S4 for the spectra). **NaBAPO** is stable for 24 h against decomposition in the presence of human plasma whereas **DoBAPO** decomposes slowly. The quotient of the **DoBAPO** integrals (relative to the triphenyl phosphate integral) post and prior incubation (0.69/0.77 = 0.90) indicates a decomposition of 10% of **DoBAPO** over 24 h. The **NaBAPO** stock solution (100 µL, 25 mM) is added to a mixture of DMEM (200 µL) and human blood plasma (200 µL, from sodium citrate (3.2%) blood) leading to a final **BAPO** concentration of 5 mM. The liposomal formulation of **DoBAPO** (50 µL, 10 mM) is added to a mixture of 1× DMEM (250 µL) and human blood plasma (200 µL, from sodium citrate (3.2%) blood) leading to a final **BAPO** concentration of 1 mM. These mixtures are added to amber NMR tubes equipped with a sealed class capillary containing triphenyl phosphate (4 mg/mL) in C_6_D_6_. The deuterium signal of C_6_D_6_ is used to lock the spectrum and the ^31^P signal of triphenyl phosphate (singlet at −17 ppm chemical shift) is used as external standard for phosphorus quantification. ^31^P NMR spectra are recorded before and after 24 h incubation at 37 °C in the dark (121.5 MHz, 256 scans for the **NaBAPO** sample and 162.0 MHz, 2k scans for the **DoBAPO** sample, apodization with 10 Hz is applied on all spectra).

### Cell Culture

MCF-7 and MDA-MB-231 cells were cultivated in DMEM medium (Fisher Thermo Scientific) with 10% FCS (Fisher Thermo Scientific) and 1% of a penicillin-streptomycin solution containing 10,000 U/mL penicillin and 10 mg/L streptomycin (Sigma Aldrich) at 5% CO_2_ and 37 °C. MCF-10A cells were cultivated in in MDCB-131 medium with 1% Glutamax, 10% fetal bovine serum (FBS), 10 ng/mL hEGF (all Fisher Thermo Scientific), 1 μg/mL hydrocortisone, and 1% of a penicillin-streptomycin solution containing 10,000 U/mL penicillin and 10 mg/L streptomycin (both Sigma-Aldrich, St. Louis, MO) at 37 °C and 5%. Cells were seeded in 96-well plates with flat bottom (Corning) and cultivated for 2–3 days until confluency.

### General procedure for the treatment of cells with BAPOs

When all cell lines had reached confluency (2–3 days), **BAPOs** (**NaBAPO** or **DoBAPO** together with Tween 20, respectively) were added at the respective concentrations with a medium change. Cells not treated with **NaBAPO** or **DoBAPO** were subjected to the respective concentration of the solvent (water or water/DPBS, respectively). After one day, the cells were washed with culture medium to remove excessive **BAPO**. Then, cells were irradiated with UV light of 365 nm (M&S Laborgeräte) for 10 min with an intensity of 210 µW/cm^2^ unless otherwise noted. For this step, the lid was removed and the distance to the cells was about 2 cm. After another day, the cells were incubated with calcein A (CalA) and ethidium homodimer-1 (EHD) to label live and dead cells (Life Technologies, Carlsbad, CA). After a few minutes, images were acquired with an ImageXpress Micro XLS plate reader (Molecular Devices) and the viability was quantified. We defined the viability as the number of living cells, displayed by green CalA fluorescence, divided by the total numbers of cells, i.e. the sum of living cells and dead cells which exhibit red nuclear fluorescence from intercalation of EHD into DNA. Each condition was measured in four independent wells.

### Image acquisition and processing

Images were acquired with an ImageXpress Micro XLS plate reader (Molecular Devices) using a 4× objective (Nikon). The exported images (tif format) were loaded into Fiji. For the quantification of the viability, in case of EHD, a threshold was applied and the number of EHD-positive nuclei was counted. In case of CalA, images were subjected to Fiji’s built-in local contrast enhancement algorithm if necessary, followed by transformation into a binary and application of Fiji’s Watershed algorithm. This was necessary due to the sometimes uneven CalA signals and the confluency of the cells. Then, cells were counted. For the colocalization analysis, the background was subtracted using the rolling ball method with a radius of ten pixels, followed by application of a Gaussian blur with a sigma of one pixel. Then, Fiji’s built-in JaCoP-Plugin was used to determine Li’s colocalization value (ICQ value).

### Colocalization analysis

To analyze the colocalization of EHD and the CellEvent signal to prove the presence of apoptosis, the ICQ value (Li’s colocalization value) is calculated which adopts values between −0.5 and +0.5. A value of −0.5 indicates exclusion or anticolocalization, 0 random colocalization and exclusion like for two images of noise, and +0.5 colocalization. It should be noted that ICQ values above 0.4 are rare in biological samples, where noise is always present.

After the standard treatment, dead cells were detected by EHD and CellEvent Caspase-3/7, a peptide-fluorophore conjugate (Life Technologies). The peptide exhibits the DEVD-amino acid sequence which is specifically cleaved by caspase 3 and 7. Upon cleavage, the dye is released and translocates into the nucleus where it binds to DNA. Hence, colocalization of the EHD and the CellEvent signal is indicative for apoptosis. The induction of apoptosis was investigated one day after UV irradiation, hence, apoptosis has progressed and the nucleus is already penetrable by EHD. Thus, we quantified colocalization following Li’s colocalization analysis^26^. Refer to the literature for a detailed explanation of ICQ analysis^27^.

### Statistics

Statistical analyses were performed with OriginPro 9.1G. Graphs show mean ± SE for every condition. As three or more groups were compared in every experiment, a one-way ANOVA followed by Bonferroni correction was performed (^*/#/§^ p ≤ 0.05, ^**/##/§§^ p ≤ 0.01, ^***/###/§§§^p ≤ 0.001). For each experiment, the compared groups are reported in the caption of the respective data plot.

## Results and Discussion

### Chemical Syntheses

**Figure 3:**
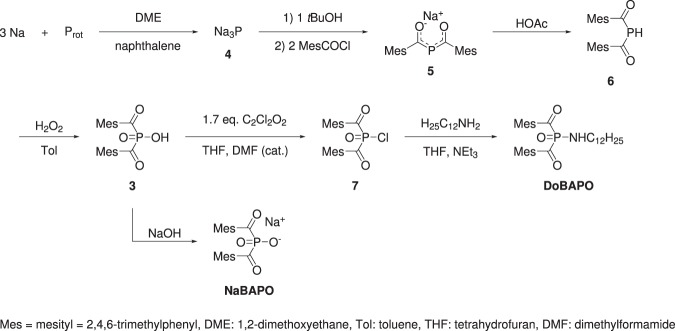
Syntheses of bismesitoylphosphinic acid (**3**)^7^, its sodium salt (**NaBAPO**), and *N*-dodecyl-*P*,*P*-bismesitoylphosphinic amide (**DoBAPO**).

The syntheses of **3** according to literature^7,25^ and the subsequent synthesis of **DoBAPO** are depicted in Fig. 3. Due to solubility reasons, **3** is converted into its sodium salt **NaBAPO** (see Figs. S1–S3 and Tables S1, S2 for characterization).

The acid **3** is converted into its chloride **7** using oxalyl chloride and dimethylformamide as nucleophilic catalyst^25^. Nucleophilic substitution with dodecylamine in presence of triethylamine to trap hydrogen chloride yields **DoBAPO**. This lipophilic derivative is applied in aqueous media together with the detergent Tween® 20 (polysorbate 20) to facilitate the formation of micelles (see Fig. S3 for characterization).

Before the compounds were tested for cellular effects, we confirmed their stability against human blood plasma under physiological conditions by quantitative ^31^P NMR spectroscopy. **NaBAPO** is completely stable over 24 h, while a minor decomposition of **DoBAPO** was observed (Fig. S4).

### NaBAPO induces cell death upon irradiation

First, we investigated if the unmodified stem compound **NaBAPO** can induce cell death in the breast cancer cell lines MCF-7 and MDA-MB-231 as well as in the non-tumorigenic breast epithelial cell line MCF-10A upon irradiation. Briefly, cells were treated with **NaBAPO** at different doses and then washed to remove excessive **NaBAPO**. One set of cells was then irradiated for 10 minutes with UV light (365 nm) at 210 µW/cm^2^ while the other set was not irradiated. Viability of the cells was determined 24 hours after irradiation with live/dead staining.

**Figure 4:**
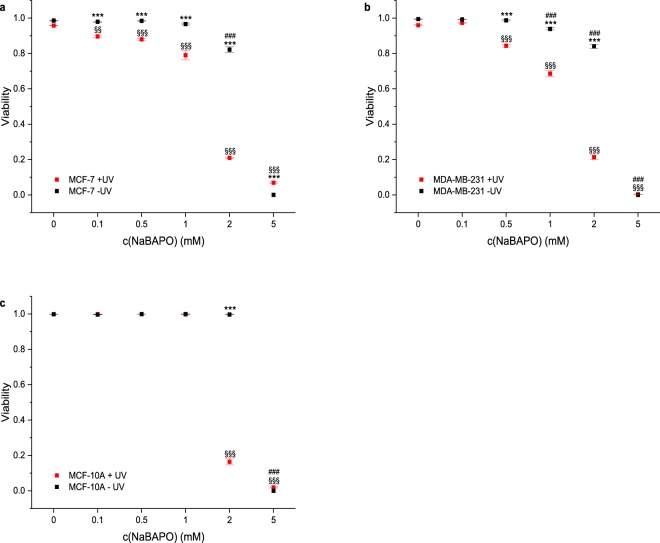
Viability of cells treated with various concentrations of **NaBAPO** and subsequently either subjected to UV irradiation or not. Cellular viability was quantified by detection of live and dead cells with calcein A and ethidium homodimer fluorescence for each cell line (n = 4). Cellular viabilities of MCF-7 cells (**a**), MDA-MB-231 cells (**b**), and MCF-10A cells (**c**) decrease significantly after **NaBAPO** treatment and UV irradiation for 10 minutes (red symbols) compared to non-**BAPO**-treated cells both subjected and not subjected to UV irradiation (red and black symbols at c = 0 mM) and non-irradiated cells (black symbols at c = 0.1 to 5 mM). Error bars: SEM. Statistical analysis was performed by one-way ANOVA followed by Bonferroni correction (***p ≤ 0.001 relative to corresponding non-irradiated condition. ^§§/§§§^p ≤ 0.01/p ≤ 0.001 relative to corresponding non-**BAPO**-treated, but irradiated control. ^#/###^p ≤ 0.05/p ≤ 0.001 relative to corresponding untreated control). See Fig. S5 for representative images.

The results of **NaBAPO** treatment of the cell lines with (+UV, red symbols) and without (−UV, black symbols) irradiation are depicted in Fig. 4 (Fig. S5 for representative images). Neither UV irradiation nor application of **NaBAPO** alone at low doses reduce cell viability. However, the combination of **NaBAPO** treatment and UV irradiation clearly decreases cell viability, a first indicator that **NaBAPO** acts as a photolatent cytotoxic agent. Quantification of the viability reveals that the tumorigenic cell lines are already significantly affected at lower concentrations (0.1, 0.5 and 1 mM), whereas the viability of MCF-10A cells is not reduced at these conditions. The decreased cell viability at the highest concentration of 5 mM without irradiation in all cell lines is regarded as unspecific due to osmotic effects as **NaBAPO** is ionic.

### The cytotoxicity of activated NaBAPO stems from the production of radicals upon irradiation

**Figure 5:**
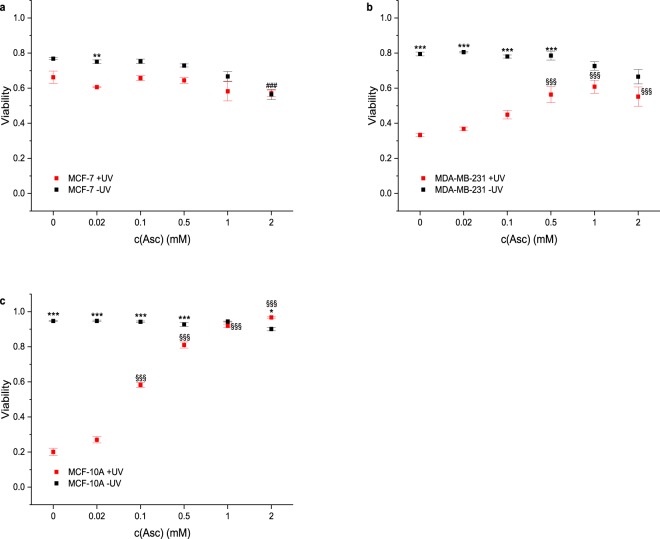
Viability of cells treated with 2 mM **NaBAPO** and various concentrations of ascorbate and subsequently either subjected to UV irradiation or not. Cellular viability was quantified as in Fig. 4 (n = 4). Cellular viability of MCF-7 cells (**a**) is unaffected by treatment with ascorbate, **NaBAPO** and 10 min UV irradiation (red symbols) compared to cells not treated with ascorbate (red and black symbols at c = 0 mM) and non-irradiated cells (black symbols at c = 0.02 to 2 mM). Cellular viabilities of MDA-MB-231 cells (**b**) and MCF-10A cells (**c**) are significantly and completely rescued by the addition of ascorbate after treatment with **NaBAPO** and 10 min UV irradiation (red symbols) compared to non-**BAPO**-treated and non-irradiated cells (black symbols). Error bars: SEM. */***p ≤ 0.001 relative to corresponding non-irradiated condition. ^§§§^p ≤ 0.001 relative to corresponding non-Asc-treated, but **NaBAPO**-treated and irradiated control. ^##^p ≤ 0.01 relative to corresponding untreated control. See Fig. S6 for representative images.

Next, we investigated the mode of action of activated **NaBAPO**. It is likely that the cytotoxicity arises from the radical production after **BAPO** activation. Thus, the presence of a radical scavenger like sodium ascorbate should rescue the cells^20,28,29^. They were cultivated as before and co-treated with 2 mM **NaBAPO**, the highest concentration that was not cytotoxic by itself (Fig. 4), and sodium ascorbate at concentrations from 0 to 2 mM (Fig. 5; representative images: Fig. S6). The experiment confirmed that co-treatment of MDA-MB-231 and MCF-10A with **NaBAPO** + UV and increasing amounts of ascorbate significantly enhanced cell viability to an almost complete rescue with 2 mM ascorbate. Non-irradiated cells showed no decrease in viability at any condition. This strongly indicates that free radicals are responsible for the cytotoxic effect. Since the radicals are only produced in the irradiated target tissue and since they are short-living, they should not affect distant parts of the organism and thus cause fewer side effects than many standard therapies. However, we did not observe the rescue for MCF-7 cells. A possible explanation might be a higher sensitivity to osmotic pressure, which might compensate for the effect of ascorbate.

### A liposome formulation of DoBAPO leads to stronger cytotoxicity at lower concentrations

**NaBAPO** shows promising characteristics concerning photoinduced cytotoxicity. However, concentrations at a millimolar level are required, probably because its ionic nature impedes cellular uptake. The uptake is likely linked to nonspecific, channel-independent processes like pinocytosis^30^. To reduce the concentration, we designed **DoBAPO**, a modified, lipophilic compound that can enter the cell by passive diffusion as it is commonly used in various transfection methods^31,32^. The poor water solubility of **DoBAPO** was overcome by the addition of polysorbate 20 leading to micelle formation as proven by dynamic light scattering (Fig. S3)^33,34^.

**Figure 6:**
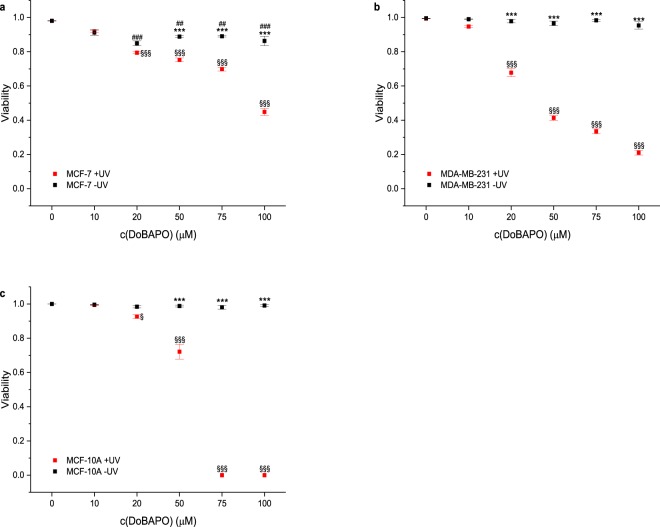
Viability of cells treated with various concentrations of **DoBAPO** and subsequently either subjected to UV irradiation or not. Cellular viability was quantified as in Fig. 4 (n = 4). Cellular viabilities of MCF-7 cells (**a**), MDA-MB-231 cells (**b**), and MCF-10 A cells (**c**) decrease significantly after treatment with **DoBAPO** and 10 min UV irradiation (red symbols) compared to non-**BAPO**-treated cells (black and red symbols at c = 0) and non-irradiated cells (black symbols). Error bars: SEM. ***p ≤ 0.001 relative to corresponding non-irradiated condition. ^§/§§§^p ≤ 0.05/p ≤ 0.001 relative to corresponding non-**BAPO**-treated, but irradiated control. ^##/###^p ≤ 0.01/p ≤ 0.001 relative to corresponding untreated control. See Fig. S7 for representative images.

Cells were treated according to the standard protocol. As expected, lower concentrations of **DoBAPO** were sufficient to induce cell death in all cell lines after UV irradiation (Fig. 6; representative images: Fig. S7). The quantification reveals a significantly lower viability at 20 μM and above compared to the non-treated yet irradiated control. The results confirm that the improved **BAPO** molecule can be used for therapeutic applications at reasonable concentrations. Since treatment of **DoBAPO** alone did not affect cell viability at any concentration, we consider the liposomal formulation to be non-toxic.

### BAPO + UV treatment induces apoptosis

Next, we wanted to clarify the exact mode of action of the **BAPO** + UV dependent cytotoxicity. Radicals are known to induce apoptotic cell death^35^, so we tested if the co-treatment with **BAPO** + UV activates caspases as an indicator for apoptosis induction.

**Figure 7:**
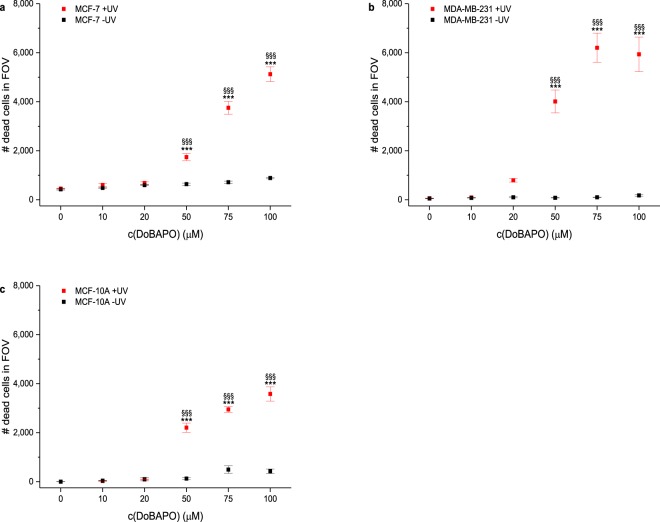
Induction of apoptosis by **DoBAPO** and subsequent UV irradiation in cells. The number of dead cells is counted as in Fig. 4 (n = 4). The number of dead MCF-7 cells (**a**), MDA-MB-231 cells (**b**), and MCF-10 A cells (**c**) increases significantly after treatment with **DoBAPO** and 10 min UV irradiation (red symbols) compared to non-**BAPO**-treated (black and red symbols at c = 0) and non-irradiated cells (black symbols). Error bars: SEM. ***p ≤ 0.001 relative to corresponding non-irradiated condition. ^§§§^p ≤ 0.001 relative to corresponding non-**BAPO**-treated, but irradiated control. See Fig. S8 for representative images.

Using a commercially available construct that fluoresces upon caspase 3/7 activity, we could show that **DoBAPO** in combination with UV irradiation visibly activates caspases 3 and 7 at a concentration of 50 μM and higher in all cell lines (Fig. S8). Counting of dead cells in the specific frames of view (FOV) confirmed that these concentrations are sufficient to significantly increase the number of dead cells compared to non-**BAPO**-treated and to non-irradiated cells (Fig. 7). As observed in previous experiments, MCF-10A cells are less affected than the other cell lines. This suggests that tumor cells might be more sensitive towards radicals than non-transformed cells, possibly due to their increased cell division rate^36,37^. However, further experiments are needed to prove if this is an exceptional effect for the investigated cell line or holds true for other cell types.

The results were supported by a colocalization between EHD and cleaved caspase 3/7 signal using the ICQ which is between 0.15 and 0.4 in all cell lines (Fig. S10; discussion of ICQ analysis in refs^26,27^). This indicates that the cells do not undergo uncontrolled necrosis which would yield low colocalization between EHD and cleaved caspase 3/7 signal, but apoptosis.

### The potency of DoBAPO depends on the irradiation dose

**Figure 8:**
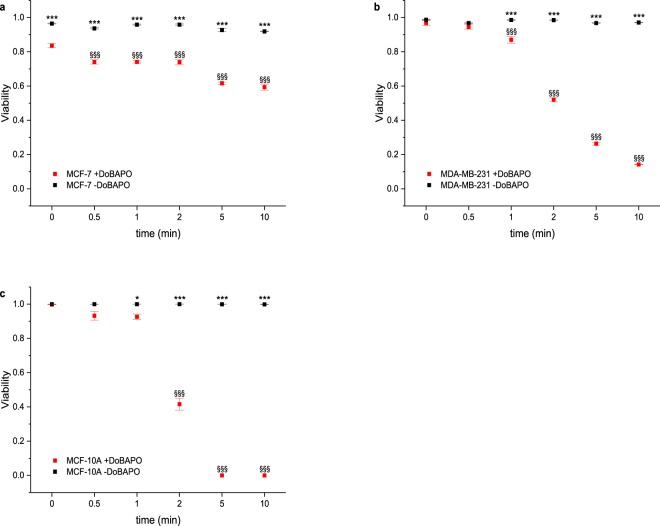
Influence of the UV irradiation dose, measured by varying the irradiation time at constant intensity, on the viability of cells treated with 100 µM **DoBAPO**. Cellular viability quantified as in Fig. 4 (n = 4). Cellular viabilities of **DoBAPO** treated MCF-7 cells (**a**), MDA-MB-231 cells (**b**), and MCF-10 A cells (**c**) decrease significantly by increased irradiation time (red symbols) compared to non-**BAPO**-treated and non-irradiated cells (black symbols at t = 0). Error bars: SEM. */***p ≤ 0.05/p ≤ 0.001 relative to corresponding non-**BAPO**-treated condition. ^§§§^p ≤ 0.001 relative to the corresponding **BAPO**-treated, but non-irradiated control. See Fig. S9 for representative images.

To investigate whether the irradiation dose influences the proapoptotic effect of **DoBAPO**, we incubated all cell lines with 100 µM **DoBAPO** according to the standard protocol. After one day, we irradiated for 0, 0.5, 1, 2, 5, and 10 min with UV light of constant intensity (210 µW/cm^2^) and cultivated the cells for another day, followed by investigation of viability. As depicted in Fig. 8, the irradiation time clearly affects the **DoBAPO** efficacy. A short irradiation time of 1–2 min already reduces the viability in all cell lines (representative images: Fig. S9). The viability is significantly decreased after an irradiation of 0.5 min in MCF-7, after 1 min in MDA-MB-231, and after 2 min in MCF-10A. In all cell lines, longer irradiation further reduces the viability. In contrast, non-**BAPO** treated cells are not affected by UV irradiation. The gradual reduction in viability indicates that not all **BAPO** molecules are cleaved by the first photons, but that the process is continuous with uncleaved **BAPO** molecules persisting. Hence, the induction of cytotoxicity cannot only be tuned by the **BAPO** concentration, but also by the irradiation dose. Again, the non-transformed MCF-10A cells are less sensitive.

The results indicate that instead of prolonging the irradiation time at a given intensity, the cells could be irradiated repetitively for short periods at lower **BAPO** concentrations. Since none of the cell lines is affected by **BAPO** or UV irradiation alone (Figs. 3 and 5), the repetitive treatment could be applied to produce radicals several times for fine-tuning a therapeutic application.

## Conclusion

In this work, we could establish **BAPOs** as a novel family of photoactivatable cytotoxic agents with potential applications as photolatent anticancer drugs. First, treatment of MCF-7, MDA-MB-231, and MCF-10A cells with **BAPOs** alone does not affect cell viability. However, upon 365 nm UV irradiation with 210 µW/cm^2^ for several minutes, apoptosis is induced. Second, the induction of apoptosis depends on both the **BAPO** dose and the irradiation dose. Finally, we could show that the cytotoxicity of the activated **BAPOs** originates from the production of radicals and not from other putative, long-lived cytotoxic by-products as cells were completely rescued from cell death in the presence of the antioxidant sodium ascorbate.

Non-irradiated **BAPOs** show low cytotoxicity up to high concentrations, equivalent to a large maximum tolerated chemical dose. However, once photoactivated, **BAPOs** act as cytotoxic agents at low concentrations, corresponding to a low minimum effective dose. These two properties of **BAPOs** would allow a variety of practicable and flexible conditions for a potential therapeutic use. This separates **BAPOs** from several conventional anticancer drugs whose adverse side reactions and narrow therapeutic windows are a constant challenge: especially older cytotoxic drugs affect fast proliferating cells in general and may attack any other cell.

To further support the approach an optimized **BAPO**-derivative **DoBAPO** was designed. This derivative enters the cell with higher efficiency leading to a stronger effect at lower doses. A possible therapeutic strategy might consist of the systemic or topic administration of **DoBAPO**, followed by repeated, short UV irradiation at the location of the target tissue, for example with an endoscopic light guide. As **DoBAPO** does not show prominent cytotoxicity, a systemic administration should be well tolerated. Radicals possess a short lifetime which is why the action of activated **BAPOs** is probably confined to the small area of UV irradiation. The tumour targeting solely arises from the local activation of **BAPOs** with light. Combined with the fact that **BAPOs** alone are non-toxic, structural variation for tissue targeting is not obligatory, but could further reduce possible side effects. Since we used UVA light (365 nm) and not UV light of higher energy, the formation of cyclobutane pyrimidine dimers is unlikely^38^. Like other cytotoxic agents, UVA light alone may however cause the formation of ROS that can induce both apoptosis and necrosis^38,39^. A long-term effect of ROS induction is the increase of protein oxidation whose accumulation triggers apoptosis^39^, a mechanism probably also underlying the **BAPO** and UVA light induced apoptosis. If UVA light alone should also affect apoptosis in our setup, we regard this as a desired side effect. However, long-term studies, e.g. concerning the formation of pyrimidine dimers, oxidized proteins or **BAPO**-related side products as well as tolerance and metabolism, are obviously needed.

Our data show that **BAPOs** represent a novel approach in anti-cancer treatment where a photoactive, non-toxic molecule itself is directly cleaved into radicals upon light irradiation. Thus, the mechanism is completely different from another approach to employ light to activate a target molecule for clinical use, the photodynamic therapy (PDT)^40^. In PDT, photosensitizers (e.g. porphyrines) or prodrugs which are metabolized into photosensitizers are administered. Once photoactivated (in most cases with low-energy red light), the exited states of the photosensitizer molecules transform cellular triplet oxygen into highly reactive singlet oxygen and other reactive oxygen species. This singlet oxygen finally induces oxidative stress and cell death. Thus, the photosensitizers used in PDT are not photo-cleaved upon activation and remain permanently active under irradiation until degraded or excreted. Therefore, a main adverse drug effect of agents applied in PDT is a light sensitivity of the treated patient, which can last over weeks^40^. In contrast, **BAPOs** and other photoinitiators are photo-cleaved upon irradiation and, within the activation process, transformed into an inactive form. Therefore, applied topically, with a sufficient light dose, the whole amount of drug can be activated at the desired area with no side effects after the treatment. The effectiveness of **BAPOs** directly correlates with the administered dose since the number of radicals being produced after irradiation is limited for every molecule. This advantage towards PDT is even more favourable since **BAPOs** can be topically administered and can therefore be cleaved at the desired area. We regard this way of administration as the most suitable method due to the low penetration of UV light. However, we would also expect less light sensitivity after systemic administration of **BAPOs** than for agents used in PDT. This is because **BAPOs** or other photoinitiators require UV or blue, energy rich light which is easier to avoid than low energy light. Alternative excitation schemes like two-photon excitation (TPA, see next paragraph) would show even lower probabilities for unwanted **BAPO** activation.

Additionally, **BAPOs** have been investigated for application in a different context for a long time which is certainly beneficial for further optimisation: established chemistry for various modifications is available, enabling the synthesis of **BAPO** species tailored for a specific clinical setting. Optical properties of **BAPOs** have been extensively characterized. Optimal excitation parameters are hence either already determined or can be easily predicted. For example, **BAPO** type photoinitiator **2** is reported in literature to be activated by TPA in high resolution 3D printing, shifting the excitation window towards the near infrared^41–44^. Even though not tested herein, the possibility of TPA even deepens the possible medical applications with respect of biocompatibility, space resolution, and penetration depth.

In summary, it is likely that a therapeutic application of **BAPOs** will be efficient while accompanied by only minimal adverse drug reactions. To tailor chemical and optical parameters, a great many of synthetic strategies is available. These features make **BAPOs** an intriguing novel class of substances for anticancer therapy.

## Supplementary information

Supplementary Information

## Data Availability

The datasets generated during and/or analysed during the current study are available from the corresponding author on reasonable request.

## References

[1] Ellrich, K. & Herzig, C. Bisacylphosphine oxide and its use. DE3443221A1 (1986).

[2] Rutsch W (1996). Recent developments in photoinitiators. Progress in Organic Coatings.

[3] Crivello, J. V. & Dietliker, K. Photoinitiators for Free Radical Cationic & Anionic Photopolymerisation. 2nd edn, Vol. III p. 168–180 (J. Wiley & Sons Ltd., SITA Technology Ltd., 1998).

[4] Dietliker, K. A Compilation of Photoinitiators Commercially available for UV Today. (SITA Technology Limited, Edinburgh and London, UK, 2002).

[5] Grützmacher H (2008). A Simple Straightforward Synthesis of Phenylphosphane and the Photoinitiator Bis(mesitoyl)phenylphosphane Oxide (IRGACURE 819). CHIMIA International Journal for Chemistry.

[6] Müller, G., Grützmacher, H. & Dietliker, K. Derivatives of bisacylphosphinic acid, their preparation and use as photoinitiators. WO2014095724A1 (2014).

[7] Müller G (2015). Simple One-Pot Syntheses of Water-Soluble Bis(acyl)phosphane Oxide Photoinitiators and Their Application in Surfactant-Free Emulsion Polymerization. Macromolecular Rapid Communications.

[8] Fast DE (2016). Bis(mesitoyl)phosphinic acid: photo-triggered release of metaphosphorous acid in solution. Chemical Communications.

[9] Potzmann PM, Lopez Villanueva FJ, Liska R (2015). UV-Initiated Bubble-Free Frontal Polymerization in Aqueous Conditions. Macromolecules.

[10] Benedikt S (2016). Highly efficient water-soluble visible light photoinitiators. Journal of Polymer Science Part A: Polymer Chemistry.

[11] Kolczak U, Rist G, Dietliker K, Wirz J (1996). Reaction Mechanism of Monoacyl- and Bisacylphosphine Oxide Photoinitiators Studied by 31P-, 13C-, and 1H-CIDNP and ESR. Journal of the American Chemical Society.

[12] Sluggett GW, McGarry PF, Koptyug IV (1996). & Turro, N. J. Laser Flash Photolysis and Time-Resolved ESR Study of Phosphinoyl Radical Structure and Reactivity. Journal of the American Chemical Society.

[13] Rees MTL, Russell GT, Zammit MD, Davis TP (1998). Visible Light Pulsed-OPO-Laser Polymerization at 450 nm Employing a Bis(acylphosphine oxide) Photoinitiator. Macromolecules.

[14] Günersel ED, Hepuzer Y, Yağcı Y (1999). Bisacylphosphine oxides as bifunctional photoinitiators for block copolymer synthesis. Die Angewandte Makromolekulare Chemie.

[15] Sluggett GW, Turro C, George MW, Koptyug IV, Turro NJ (1995). (2, 4, 6-Trimethylbenzoyl)diphenylphosphine Oxide Photochemistry. A Direct Time-Resolved Spectroscopic Study of Both Radical Fragments. Journal of the American Chemical Society.

[16] Kleinpenning MM (2010). Clinical and histological effects of blue light on normal skin. Photodermatology, Photoimmunology & Photomedicine.

[17] Opländer C (2011). Effects of blue light irradiation on human dermal fibroblasts. Journal of Photochemistry and Photobiology B: Biology.

[18] Kannan K, Jain SK (2000). Oxidative stress and apoptosis. Pathophysiology.

[19] Chandra J, Samali A, Orrenius S (2000). Triggering and modulation of apoptosis by oxidative stress. Free Radical Biology and Medicine.

[20] Winterbourn CC (2008). Reconciling the chemistry and biology of reactive oxygen species. Nat Chem Biol.

[21] Trachootham D, Alexandre J, Huang P (2009). Targeting cancer cells by ROS-mediated mechanisms: a radical therapeutic approach?. Nat Rev Drug Discov.

[22] Sigma-Aldrich Chemie GmbH: Phenylbis (2,6-trimethylbenzoyl) phosphine oxide, MSDS No. 511447 (Online), 23.07.2015, accessed 14.04.2016.

[23] European Chemicals Agency: Phenyl bis(2,6-trimethylbenzoyl)-phosphine oxide, https://echa.europa.eu/en/substance-information/-/substanceinfo/100.102.189, accessed 13.09.2016.

[24] Dolomanov OV, Bourhis LJ, Gildea RJ, Howard JAK, Puschmann H (2009). OLEX2: a complete structure solution, refinement and analysis program. Journal of Applied Crystallography.

[25] Müller, G. S. Phosphorus Based Photoinitiators: Synthesis and Application, ETH Zürich (2013).

[26] Li Q (2004). A Syntaxin 1, Gαo, and N-Type Calcium Channel Complex at a Presynaptic Nerve Terminal: Analysis by Quantitative Immunocolocalization. The Journal of Neuroscience.

[27] Vater, M. *et al*. New insights into the intracellular distribution pattern of cationic amphiphilic drugs. **7**, 44277, 10.1038/srep44277 (2017). 10.1038/srep44277 PMC5345070 28281674

[28] Cort WM (1974). Antioxidant activity of tocopherols, ascorbyl palmitate, and ascorbic acid and their mode of action. Journal of the American Oil Chemists Society.

[29] Forrest VJ, Kang Y-H, McClain DE, Robinson DH, Ramakrishnan N (1994). Oxidative stress-induced apoptosis prevented by trolox. Free Radical Biology and Medicine.

[30] Doherty GJ, McMahon HT (2009). Mechanisms of Endocytosis. Annual Review of Biochemistry.

[31] Sercombe, L. et al. Advances and Challenges of Liposome Assisted Drug Delivery. Frontiers in Pharmacology **6**, 10.3389/fphar.2015.00286 (2015). 10.3389/fphar.2015.00286 PMC4664963 26648870

[32] Zylberberg C, Matosevic S (2016). Pharmaceutical liposomal drug delivery: a review of new delivery systems and a look at the regulatory landscape. Drug Delivery.

[33] Ismail AA, Gouda MW, Motawi MM (1970). Micellar solubilization of barbiturates I: Solubilities of certain barbiturates in polysorbates of varying hydrophobic chain length. Journal of Pharmaceutical Sciences.

[34] Kerwin BA (2008). Polysorbates 20 and 80 used in the formulation of protein biotherapeutics: Structure and degradation pathways. Journal of Pharmaceutical Sciences.

[35] Dröge W (2002). Free Radicals in the Physiological Control of Cell Function. Physiological Reviews.

[36] Hanahan D, Weinberg RA (2000). The Hallmarks of Cancer. Cell.

[37] Hanahan D, Weinberg RA (2011). Hallmarks of Cancer: The Next Generation. Cell.

[38] Ryter SW (2006). Mechanisms of Cell Death in Oxidative Stress. Antioxidants & Redox Signaling.

[39] Driscoll CO, Cotter TG (2006). ROS and protein oxidation in early stages of cytotoxic drug induced apoptosis AU - England, Karen. Free Radical Research.

[40] Dolmans DEJGJ, Fukumura D, Jain RK (2003). Photodynamic therapy for cancer. Nat Rev Cancer.

[41] Belfield KD (2000). Multiphoton-absorbing organic materials for microfabrication, emerging optical applications and non-destructive three-dimensional imaging. Journal of Physical Organic Chemistry.

[42] Belfield, K. D. & Schafer, K. J. In Photoinitiated Polymerization Vol. 847 ACS Symposium Series Ch. 39, 464–481 (American Chemical Society, 2003).

[43] Schafer KJ (2004). Two-photon absorption cross-sections of common photoinitiators. Journal of Photochemistry and Photobiology A: Chemistry.

[44] Mueller JB, Fischer J, Mange YJ, Nann T, Wegener M (2013). *In-situ* local temperature measurement during three-dimensional direct laser writing. Applied Physics Letters.

