# Supplementary material for: Bisacylphosphane oxides as photo-latent cytotoxic agents and potential photo-latent anticancer drugs
Source: Sci Rep. 2019 Apr 12;9:6003. doi: 10.1038/s41598-019-42026-y (PMC6461680; doi:10.1038/s41598-019-42026-y)
Supplement: Supplementary file 1 — Supplementary Information [file 41598_2019_42026_MOESM1_ESM.docx]

Bisacylphosphane oxides as photo-latent cytotoxic agents and potential photo-latent anticancer drugs

*Supporting Information*

Andreas Beil, Friederike A. Steudel, Christoph Bräuchle, Hansjörg Grützmacher, Leonhard Möckl

Contents

[Chemical analysis of **DoBAPO** 2](#_Toc536358968)

[Toxicological data of phenylbismesitoylphosphine oxide (**2**) and sodium bismesitoylphosphinate (NaBAPO) 4](#_Toc536358969)

[Determination of particles size in liposomal formulation of **DoBAPO** 6](#_Toc536358970)

[Plasma stability of BAPOs 7](#_Toc536358971)

[Representative Images of *in vitro* Studies 8](#_Toc536358972)

[Colocalization analysis 13](#_Toc536358973)

[Representative images for colocalization analysis 14](#_Toc536358974)

[Supplementary References 14](#_Toc536358975)

# Chemical analysis of **DoBAPO**

**^1^H NMR** (400 MHz, CDCl_3_, 298 K) δ = 6.89 (s, 4H, Mes-*H*), 3.18 (dt, *J*_PH_ = 13.5 Hz, *J*_HH_ = 6.7 Hz, 1H, N*H*), 2.86 (pseudo-quint, *J*_HH_ = 10.7 Hz, *J*_PH_/*J*_HH_ = 7.1 Hz, 2H, NHC*H*_2_(CH_2_)_10_), 2.36 (s, 12H, o-Mes-C*H*_3_), 2.31 (s, 6H, p-Mes-C*H*_3_); 1.40-1.10 (m, 20H, CH_2_(C*H*_2_)_10_CH_3_), 0.91 (t, *J*_HH_ = 6.8 Hz, 3H, CH_2_(CH_2_)_10_C*H*_3_); **^13^C{^1^H} NMR** (100.6 MHz, CDCl_3_, 298 K) δ = 218.1 (d, *J*_PC_ = 86.1 Hz, P*C*O), 140.8 (p-Mes), 136.2 (d, *J*_PC_ = 43.5 Hz, ipso-Mes), 135.5 (o-Mes), 129.0 (m-Mes), 40.0 (d, *J*_PC_ = 2.5 Hz, NH*C*H_2_(CH_2_)_10_), 31.1 (d, *J*_PC_ = 4.2 Hz, NHCH_2_*C*H_2_(CH_2_)_9_), 31.9 (*C*H_2_), 29.6(4) (*C*H_2_), 29.5(6) (*C*H_2_), 29.5 (*C*H_2_), 29.4 (*C*H_2_), 29.2 (*C*H_2_), 26.5 (*C*H_2_), 22.7 (*C*H_2_), 21.2 (p-Mes-*C*H_3_), 19.7 (o-Mes-*C*H_3_); 14.1 ((CH_2_)_10_*C*H_3_); **^31^P NMR** (162.0 MHz, CDCl_3_, 298 K) δ = 2.51 (dt, *J*_PH_ = 13.5 Hz, *J*_PH_ = 7.2 Hz); **FT-IR** ν/cm^-1^ = 3112 w, 2919 m, 2852 m, 1720 w, 1668 m, 1646 m, 1608 m, 1567 w, 1469 w, 1444 w, 1423 m, 1378 w, 1299 w, 1280 w, 1215 w, 1196 s, 1149 m, 1101 m, 1087 w, 1063 w, 1034 m, 1004 w, 963 m, 936 w, 918 m, 882 w, 846 s, 806 w, 767 w, 734 m, 720 m, 686 m, 649 w, 618 m; **Rf** (Silica, EtOAc) = 0.92; **EA** (calc.) 73.11 % C, 9.20 % H, 2.66 % N; **EA** (meas.) 73.08 % C, 9.36 % H, 2.68 % N; **HRMS** (MALDI) m/z calc. for C_32_H_49_NO_3_P^+^ (M+H^+^) 526.3445, found 526.3442, m/z calc. for C_32_H_48_NNaO_3_P^+^ (M+Na^+^) 548.3264, found 548.3261, m/z calc. for C_32_H_48_KNO_3_P^+^ (M+K^+^) 564.3003, found 564.3001, m/z calc. for C_64_H_94_N_2_NaO_6_P_2_^+^ (2 M+Na^+^) 1073.6636, found 1073.6627; Θ_m_ = 84.5-85.1 °C.


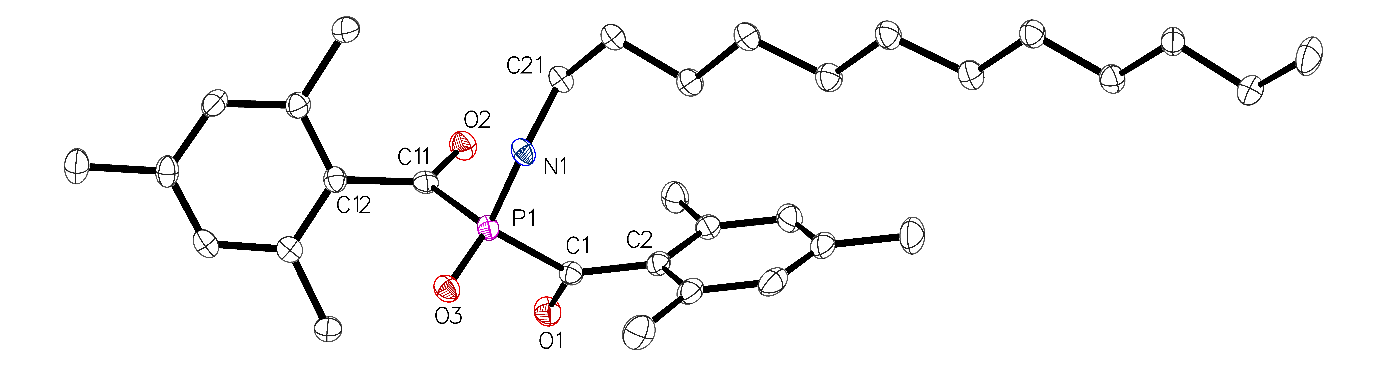


Figure S 1 Molecular structure of **DoBAPO** (thermal ellipsoids at 50 % probability, H atoms are omitted for clarity). Selected bond distances (Å), angles and torsion angles (°): P1-N1 1.619(1), P1-O3 1.478(1), C1-O1 1.213(2), C11-O2 1.219(2), P1-C1 1.875(2) ,P1-C11 1.860(2), O3-P1-N1 113.59(7), O3-P1-C1 112.20(7), O3-P1-C11 115.48(7), O1-C1-P1 114.0(1), O2-C11-P1 115.5(1), N1-P1-C1 109.49(7), N1-P1-C11 105.02(7), C1-P1-C11 100.00(7), O1-C1-P1-O3 75.7(1), O2-C11-P1-O3 -165.2(1), O1-C1-P1-N1 -157.2(1), O2-C11-P1-N1 68.8(1).

Table S 1 Crystal data and structure refinement parameters for **DoBAPO**

| CCDC number | 1503437 |
| --- | --- |
| Empirical formula | C_32_H_48_NO_3_P |
| Formula weight | 525.68 |
| Temperature/K | 100 |
| Crystal system | triclinic |
| Space group | P-1 |
| a/Å | 8.1283(2) |
| b/Å | 11.8852(3) |
| c/Å | 16.8586(4) |
| α/° | 72.9720(10) |
| β/° | 86.5360(10) |
| γ/° | 79.9610(10) |
| Volume/Å^3^ | 1533.33(7) |
| Z | 2 |
| ρ_calc_g/cm^3^ | 1.139 |
| μ/mm^‑1^ | 0.121 |
| F(000) | 572.0 |
| Crystal size/mm^3^ | 0.329 × 0.279 × 0.184 |
| Radiation | MoKα (λ = 0.71073) |
| 2Θ range for data collection/° | 2.526 to 58.596 |
| Index ranges | -11 ≤ h ≤ 11, -16 ≤ k ≤ 16, -23 ≤ l ≤ 23 |
| Reflections collected | 33488 |
| Independent reflections | 8352 [R_int_ = 0.0455, R_sigma_ = 0.0629] |
| Data/restraints/parameters | 8352/0/345 |
| Goodness-of-fit on F^2^ | 1.047 |
| Final R indexes [I>=2σ (I)] | R_1_ = 0.0554, wR_2_ = 0.1295 |
| Final R indexes [all data] | R_1_ = 0.0820, wR_2_ = 0.1421 |
| Largest diff. peak/hole / e Å^-3^ | 0.45/-0.33 |

# Toxicological data of phenylbismesitoylphosphine oxide (**2**) and sodium bismesitoylphosphinate (NaBAPO)

Phenylbismesitoylphosphine oxide Irgacure® 819 (**2**) has the largest economical relevance of all BAPO type photoinitiators. Between 100 and 1000 tons are produced or imported in the European Economic Area per year. Therefore, it has been fully evaluated according to REACH (Regulation concerning the Registration, Evaluation, Authorisation and Restriction of Chemicals (REACH), establishing a European Chemicals Agency (ECHA)) and found to be non-toxic (listed as “H317: May cause an allergic skin reaction” and “H413: May cause long-lasting harmful effects to aquatic life”).[^1^](#_ENREF_1) The safety data sheet of commercial supplier Sigma-Aldrich[^2^](#_ENREF_2) reports toxicological data as follows: LD_50_ (oral, rat) > 2 g/kg, LD_50_ (dermal, rat) > 2 g/kg, no irritant effect after 4 h (dermal, rabbit), no irritant effect after 72 h (eye, rabbit), skin sensitization possible (guinea pig maximization test positive), no germ cell mutagenicity (Ames test negative), no carcinogenicity dues to IARC.

For **NaBAPO**, reference[^3^](#_ENREF_3) reports a LC_50_ of 2.8 mM on mouse fibroblast cells L929.

UV-Vis spectra and molar extinction coefficients

Figure S 2 UV-Vis spectra of **NaBAPO** in PBS (A), the liposomal formulation consisting of **DoBAPO** (B) and Tween® 20 and **DoBAPO** in EtOH (C). The inset in B shows the full UV-Vis range.

Table S 2 UV-Vis absorption data of **NaBAPO**, **DoBAPO/Tween** and **DoBAPO**

| Compound | Solvent | λ_max_/nm | ε(λ_max_)/Lmol^-1^cm^-1^ | | ε(365 nm)/Lmol^-1^cm^-1^ | |
| --- | --- | --- | --- | --- | --- | --- |
| **NaBAPO** | PBS | 284 | 3924±126 | R^2^ = 99.7 % | 255±77 | R^2^ = 76.8 % |
| **DoBAPO/Tween** | PBS | 284 | 8248±294 | R^2^ = 99.6 % | 4540±52 | R^2^ = 99.99 % |
| **DoBAPO** | EtOH | 286 | 6150±122 | R^2^ = 99.9 % | 300±43 | R^2^ = 94.2% |

# Determination of particles size in liposomal formulation of **DoBAPO**





Figure S 3 Particle size distribution of liposomal formulation of **DoBAPO** with Tween® 20 in PBS at 10 µM, 25 µM, 50 µM and 100 µM concentration. At all concentrations, the particles sizes are found to be equally distributed around 35 nm. Error bars indicate standard errors (n = 3).

# Plasma stability of BAPOs


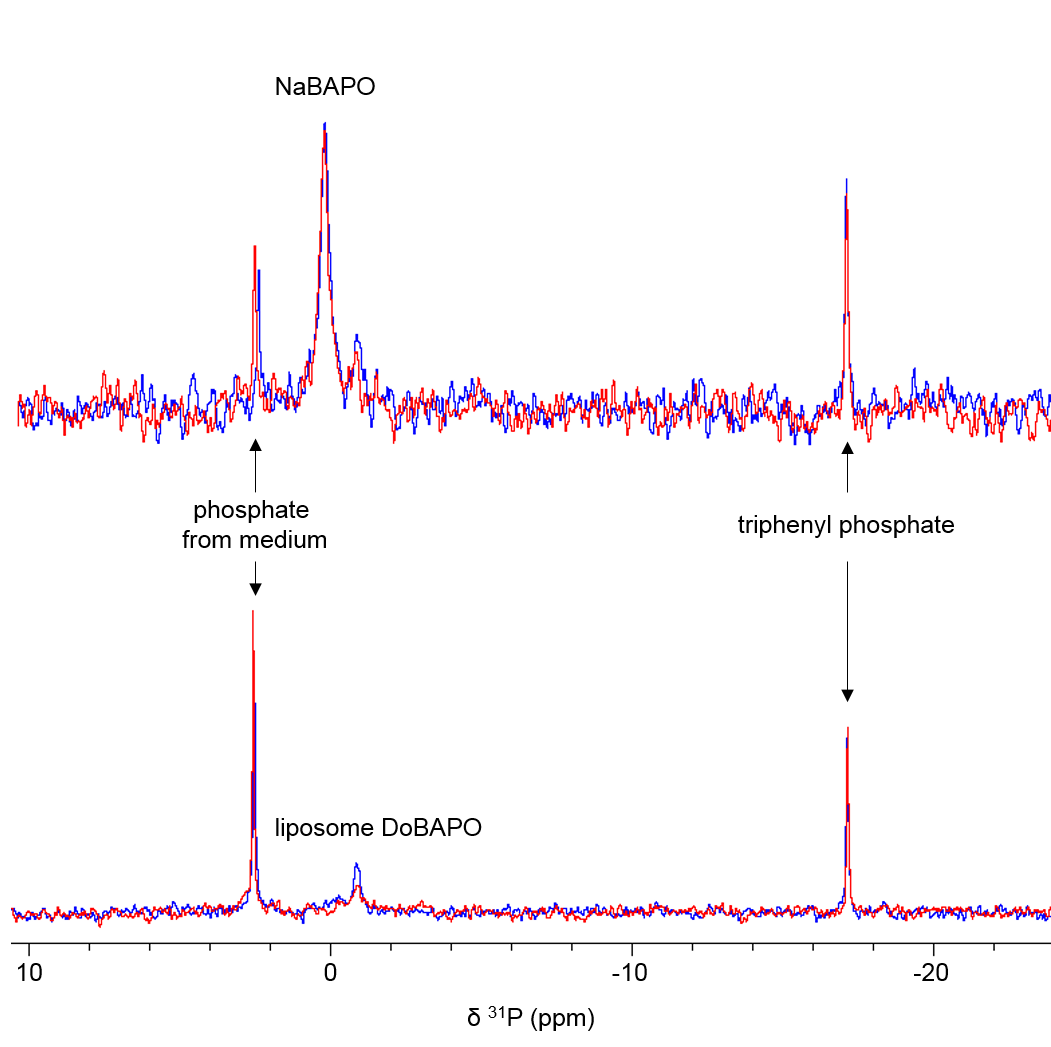


Figure S 4 ^31^P NMR spectra of BAPOs recorded prior (blue) and after (red) incubation for 24 h at 37 °C in the presence of human plasma. Top: **NaBAPO** in PBS and human plasma. Bottom: Liposomal formulation of DoBAPO in PBS and human plasma. Note the perfect overlay of both spectra for **NaBAPO** (top) while some decomposition of **DoBAPO** is observed (bottom). The signals at ‑17 ppm originate from triphenyl phosphate as external standard for ^31^P quantification. The signals around 2.5 ppm are assigned to phosphates contained in DMEM medium and plasma (data not shown). Apodization with 10 Hz is applied on all spectra.

# Representative Images of *in vitro* Studies

Figure S 5 Viability of MCF-7, MDA-MB-231, and MCF-10A cells treated with various concentrations of **NaBAPO** and subsequently either subjected to UV irradiation or not. Cellular viability was quantified by detection of live and dead cells with calcein A (cyan) and ethidium homodimer (magenta) fluorescence for each cell line (n = 4-5). Representative images of MCF-7 cells (**a**), MDA-MB-231 cells (**b**), and MCF-10A cells (**c**) treated with **NaBAPO** ± UV for 10 min. Scale bar: 1 mm.

Figure S 6 Viability of MCF-7, MDA-MB-231, and MCF-10A cells treated with 2 mM **NaBAPO** and various concentrations of ascorbate and subsequently either subjected to UV irradiation or not. Cellular viability was quantified by detection of live and dead cells with calcein A (cyan) and ethidium homodimer (magenta) fluorescence for each cell line (n = 4). Representative images of MCF-7 cells (**a**), MDA-MB-231 cells (**b**), and MCF-10A cells (**c**) treated with **NaBAPO** ± UV for 10 min. Scale bar: 1 mm.

Figure S 7 Viability of MCF-7, MDA-MB-231, and MCF-10A cells treated with various concentrations of **DoBAPO** and subsequently either subjected to UV irradiation or not. Cellular viability was quantified by detection of live and dead cells with calcein A (cyan) and ethidium homodimer (magenta) fluorescence for each cell line (n = 4). Representative images of MCF-7 cells (**a**), MDA-MB-231 cells (**b**), and MCF-10 A cells (**c**) treated with **DoBAPO** ± UV for 10 min. Scale bar: 1 mm.

Figure S 8 Induction of apoptosis by **DoBAPO** and subsequent UV irradiation in MCF-7, MDA-MB-231, and MCF-10A cells. The number of dead cells is counted by caspase (cyan) and EHD (magenta) signal for each cell line (n = 4). Representative images of MCF-7 cells (**a**), MDA-MB-231 cells (**b**), and MCF-10 A cells (**c**) treated with **DoBAPO** ± UV for 10 min. Scale bar: 1 mm.

Figure S 9 Influence of the UV irradiation dose, measured by varying the irradiation time at constant intensity, on the viability MCF-7, MDA-MB-231, and MCF-10A cells treated with 100 µM **DoBAPO**. Cellular viability was quantified by detection of live and dead cells with calcein A (cyan) and ethidium homodimer (magenta) fluorescence for each cell line (n = 4). Representative pictures of MCF-7 cells (**a**), MDA-MB-231 cells (**b**), and MCF-10 A cells (**c**) with UV irradiation ± 100 µM **DoBAPO** for the indicated time. Scale bar: 1 mm.

# Colocalization analysis





Figure S 10 Induction of apoptosis by **DoBAPO** and subsequent UV irradiation by determination of ICQ.[^4^](#_ENREF_4) Colocalization between EHD signal and CellEvent™ caspase 3/7 signal in MCF-7 cells (**a**), MCF-10A cells (**b**) and MDA-MB-231 cells (**c**). Clearly, EHD and CellEvent™ colocalize over the whole applied concentration range, both for irradiated and not irradiated cells. This indicates that dying, EHD positive cells are not necrotic, but apoptotic. Decreased ICQ around zero can be attributed to low numbers of dead cells and hence increasing contribution of random noise. Error bars: SEM. Statistical analysis was performed by one-way ANOVA followed by Bonferroni correction (***: p≤0.001 relative to corresponding non-irradiated condition. §§§: p≤0.001 relative to corresponding non-BAPO-treated, but irradiated control. ##: p≤0.01 relative to corresponding untreated control).

# Representative images for colocalization analysis


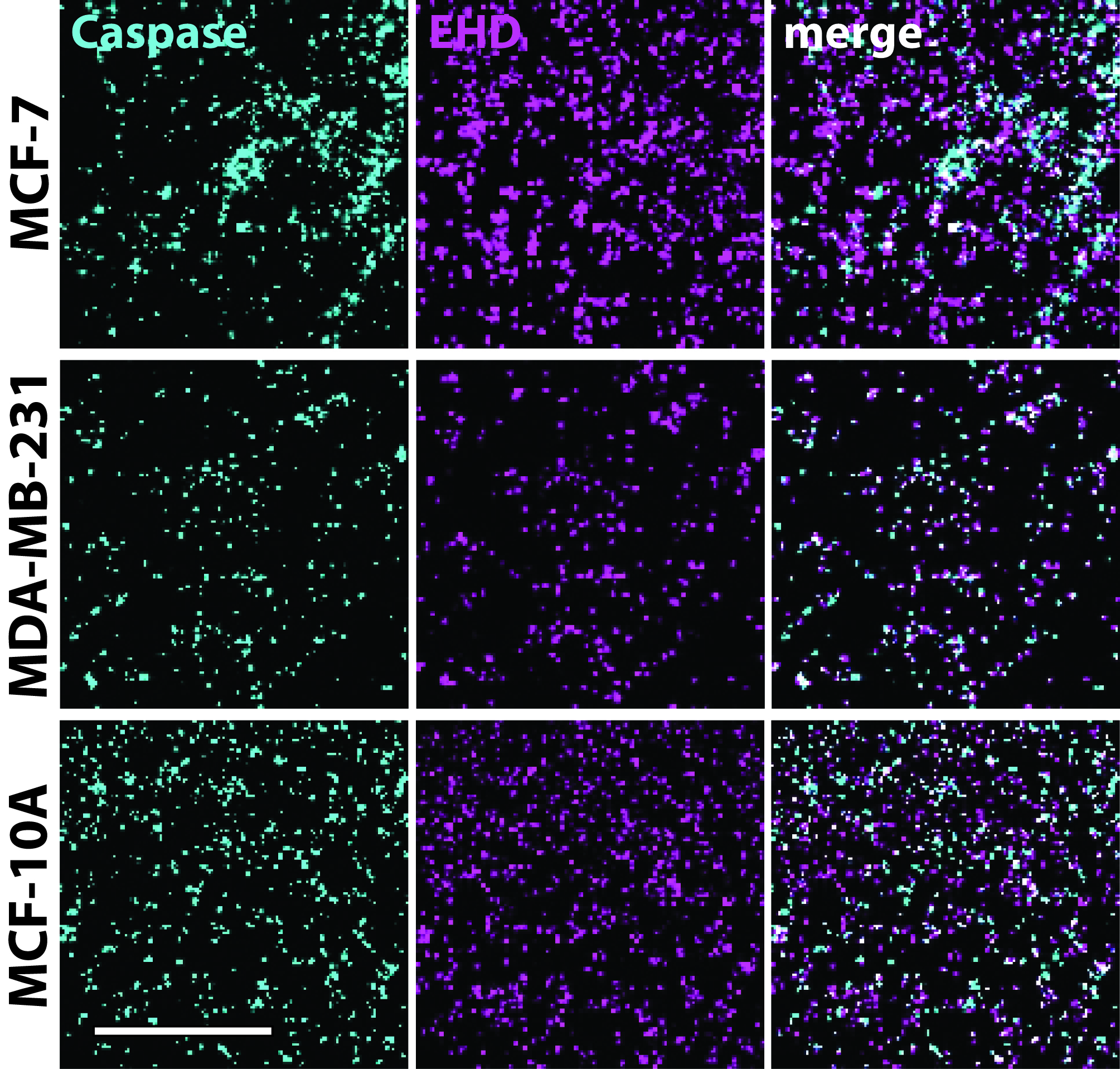


Figure S 11 Representative images of Caspase/EHD signal colocalization for 100 µM DoBAPO and UV treatment. Scale bar: 0.5 mm.

# Supplementary References

1 European Chemicals Agency, Phenyl bis(2,4,6-trimethylbenzoyl)-phosphine oxide, <https://echa.europa.eu/en/substance-information/-/substanceinfo/100.102.189>, accessed 13.09.2016

2 Sigma-Aldrich Chemie GmbH, CH-9471 Buchs, Phenylbis(2,4,6-trimethylbenzoyl)phosphine oxide, MSDS No. 511447 (Online), 23.07.2015, accessed 14.04.2016

3 Benedikt, S. *et al.* Highly efficient water-soluble visible light photoinitiators. *Journal of Polymer Science Part A: Polymer Chemistry* **54**, 473-479, doi:10.1002/pola.27903 (2016).

4 Li, Q. *et al.* A Syntaxin 1, Gα_o_, and N-Type Calcium Channel Complex at a Presynaptic Nerve Terminal: Analysis by Quantitative Immunocolocalization. *The Journal of Neuroscience* **24**, 4070-4081, doi:10.1523/jneurosci.0346-04.2004 (2004).
